# The Effects of Indoxyl Sulfate and Oxidative Stress on the Severity of Peripheral Nerve Dysfunction in Patients with Chronic Kidney Diseases

**DOI:** 10.3390/antiox11122350

**Published:** 2022-11-28

**Authors:** Yun-Ru Lai, Ben-Chung Cheng, Chia-Ni Lin, Wen-Chan Chiu, Ting-Yin Lin, Hui-Ching Chiang, Chun-En Aurea Kuo, Chih-Cheng Huang, Cheng-Hsien Lu

**Affiliations:** 1 Department of Neurology, Kaohsiung Chang Gung Memorial Hospital, Chang Gung University College of Medicine, Kaohsiung 83301, Taiwan; 2 Department of Hyperbaric Oxygen Therapy Center, Kaohsiung Chang Gung Memorial Hospital, Chang Gung University College of Medicine, Kaohsiung 83301, Taiwan; 3 Department of Internal Medicine, Kaohsiung Chang Gung Memorial Hospital, Chang Gung University College of Medicine, Kaohsiung 83301, Taiwan; 4 Department of Laboratory Medicine, Chang Gung Memorial Hospital Lin-Kou Branch, Chang Gung University, Taoyuan 83301, Taiwan; 5 Department of Nursing, Kaohsiung Chang Gung Memorial Hospital, Chang Gung University College of Medicine, Kaohsiung 83301, Taiwan; 6 Department of Chinese Medicine, Kaohsiung Chang Gung Memorial Hospital, Chang Gung University College of Medicine, Kaohsiung 83301, Taiwan; 7 Center for Shockwave Medicine and Tissue Engineering, Kaohsiung Chang Gung Memorial Hospital, Chang Gung University College of Medicine, Kaohsiung 83301, Taiwan; 8 Department of Biological Science, National Sun Yat-Sen University, Kaohsiung 80424, Taiwan; 9 Department of Neurology, Xiamen Chang Gung Memorial Hospital, Xiamen 361126, China

**Keywords:** chronic kidney disease, indoxyl sulfate, oxidative stress, protein-binding uremic toxin, severity of peripheral nerve dysfunction

## Abstract

Pieces of evidence support the view that the accumulation of uremic toxins enhances oxidative stress and downstream regulation of signaling pathways, contributing to both endothelial microangiography and cell dysfunction. This study is to address the impact of protein-binding uremic toxins on the severity of peripheral nerve function in patients with chronic kidney disease (CKD). Fifty-four patients with CKD were included in the Toronto Clinical Neuropathy Score (TCNS), nerve conduction study (NCS), and laboratory studies including protein-binding uremic toxin (indoxyl sulfate [IS] and *p*-cresyl sulfate [PCS]), oxidative stress (Thiol and thiobarbituric acid reacting substances [TBARS]), and endothelial dysfunction (serum intercellular adhesion molecule 1 [sICAM-1] and serum vascular adhesion molecule 1 [sVCAM-1]) at enrollment. We used composite amplitude scores (CAS) to analyze the severity of nerve conductions on peripheral nerve function. TCNS and CAS were higher in the diabetic CKD group (*p* = 0.02 and 0.01, respectively). The NCS revealed the compound muscle action potential of ulnar and peroneal nerves and the sensory nerve action potential of ulnar and sural nerves (*p* = 0.004, *p* = 0.004, *p* = 0.004, and *p* = 0.001, respectively), which was found to be significantly low in the diabetic group. CAS was significantly correlated with age (r = 0.27, *p* = 0.04), urine albumin-creatinine ratio (UACR) (r = 0.29, *p* = 0.046), free-form IS (r = 0.39, *p* = 0.009), sICAM-1 (r = 0.31, *p* = 0.02), sVCAM-1 (r = 0.44, *p* < 0.0001), TBARS (r = 0.35, *p* = 0.002), and thiols (r = −0.28, *p* = 0.045). Linear regression revealed that only TBARS and free-form IS were strongly associated with CAS. The mediation analysis shows that the sVCAM-1 level serves as the mediator between higher IS and higher CAS. IS and oxidative stress contribute to the severity of peripheral nerve dysfunction in patients with CKD, and chronic glycemic impairment can worsen the conditions.

## 1. Introduction

Chronic kidney disease (CKD) remains a significant global burden [1]. The prevalence rate of CKD is at around 5–15% among adults, and the prevalence increases with age [2]; the global all-age prevalence has increased by 29.3% since 1990 [1]. It is also an important risk factor for morbidity and mortality in cardiovascular disease [1]. Neurological complications may potentially affect both the central and peripheral nervous systems of patients in the pre-dialysis and dialysis stages [3]. Peripheral nerve function impairment in CKD is a common neurological complication that affects 90% of dialysis patients [4,5]. In contrast, patients with CKD who had type 2 diabetes mellitus (T2DM) develop neuropathy earlier, especially in the third stage of CKD [6] or even when T2DM is diagnosed [7]. The common presentation of peripheral neuropathy in non-diabetic CKD is similar to T2DM, a slowly progressive sensorimotor polyneuropathy that begins in the feet and legs and may spread to the hands and arms [4,5,7].

Evidence supports the view that intestinal dysbiosis induced by an imbalanced or/and excess of nutrients leads to overproduction and accumulation of uremic retention solutes (URS) (e.g., indoxyl sulfate [IS], p-cresyl sulfate [PCS], and urea) in the intestine [8]. The gradual decline in renal function prevents the kidneys from clearing the URS (called uremic toxins) normally and is responsible for uremic syndrome [8]. Further, metabolomic studies showed that URS is associated with perturbations of glucose homeostasis even in the absence of diabetes [9]. IS and PCS, the two most well-investigated protein-bound uremic toxins, can enhance the nuclear factor-kappa B (NF-kB) pathway, resulting in the production of pro-inflammatory cytokine and oxidative stress in renal tubular cells, causing cell damage. The IS also induces free radicals’ production, which leads to harm to the vascular endothelial cells [10].

The mechanism of peripheral nerve dysfunction in CKD could be multifactorial [11,12]. Previous studies revealed that electrolyte imbalance (e.g., hyperkalemia and hyperphosphatemia) can cause chronic uremic depolarization of nerves and that maintenance of serum potassium levels within normal limits between periods of dialysis is likely to reduce the severity of peripheral nerve function [12]. Besides the role of electrolyte imbalance, it is currently believed that the long-term accumulation of protein-bound uremic toxins could contribute to the generation of oxidative stress and endothelial dysfunction, followed by nerve damage. Currently, there is no promising effective treatment for the improvement of peripheral nerve function in CKD. Some data confirmed that adequate glycemic control and individualized cardiometabolic targets are the only means of minimizing the risk of peripheral neuropathy of T2DM [13].

Regarding the research on protein-bound uremic toxins, most studies focus on CKD [14,15] or its association with cardiovascular events [16]. Besides protein-bound uremic toxin, chronic glycemic impairment seems to have more deleterious effects, such as inducing oxidative stress and promoting the development of peripheral neuropathy [17]. To address the prognostic value of protein-bound uremic toxins and the effects of chronic glycemic impairment on the severity of peripheral neuropathy, we have tested the hypotheses that protein-bound uremic toxins are strongly associated with the severity of peripheral nerve function in patients with CKD and that chronic glycemic impairment can worsen the conditions.

## 2. Patients and Methods

A total of 54 patients with pre-dialysis CKD (CKDs stage 3B-4) who visited the nephrology outpatient clinic at Kaohsiung Chang Gung Memorial Hospital in Taiwan were recruited for this study. CKD is defined as decreased kidney function that persists for more than 3 months, and all enrolled patients were regularly followed up for more than 6 months (Table 1). The exclusion criteria included lumbosacral radiculopathy and alcoholic polyneuropathy. All participants understood the purpose of this study and signed an informed consent form.

### 2.1. Peripheral Nerve Function Assessment

All patients underwent complete neurologic examinations, nerve conduction studies (NCS), Toronto Clinical Neuropathy Score (TCNS), electrochemical skin conductance (ESC), as well as biochemical testing including protein-bound uremic toxin. TCNS, which consists of large and small fiber evaluations, is a valid instrument that reflects the presence and severity of a wide spectrum of polyneuropathies [18]. Baseline demographic data, underlying diseases, and laboratory parameters were obtained at baseline. The assessment of urine albumin-creatinine ratio (UACR) and estimated glomerular filtration rate (eGFR) [19,20], diabetic retinopathy (DR) [21], and vascular risk factors, including hypertension and dyslipidemia, were according to the criteria of a previous study [22].

The NCS was performed using Nicolet Viking machines (Madison, Wisconsin). The recordings for the attributes measured in each nerve included distal latency, amplitude, and nerve conduction velocity (NCV), according to our previously published paper [23]. All data obtained were compared with reference values from our laboratory [23]. The sensory and motor nerves on the bilateral side were tested, and only the ones on the non-dominant side were recorded. The severity of peripheral nerve function was based on CAS of nerve conduction, which was composed of peroneal compound muscle action potential (CMAP), tibial CMAP, ulnar CMAP, sural sensory nerve action potential (SNAP), and ulnar SNAP amplitudes. These percentile values were expressed as points from obtained percentile values according to our previously published paper, and the five attributes of NCS provided a scale from 0 to 10 points [22]. Sudoscan (Impeto Medical, Paris, France) is a non-invasive electrochemical skin conductance (ESC) device for the assessment of small fibers innervating sweat glands that enables the assessment of the function of small fibers. ESC was performed with the patients placing their hands and feet on electrode plates with a low-voltage current (<4 V) for 3 mins [24]. The values of ESC for the hands and feet were generated from the derivative current associated with the applied voltage.

### 2.2. Measurements of Biomarkers for Oxidative Stress, Endothelial Dysfunction, and Protein-Binding Uremic Toxins

We measured the serum thiobarbituric acid-reactive substance (TBARS) for oxidative stress, while the serum level of total reduced thiols was measured for anti-oxidative capacity in response to the increased oxidative damage. Serum TBARS levels were measured using a commercially available assay kit (Cayman Chemical, Ann Arbor, MI, USA, cat. no. 10009055) [25]. The serum levels of serum intercellular adhesion molecule 1 (sICAM-1) and serum vascular adhesion molecule 1 (sVCAM-1) were evaluated as the biomarkers for endothelial dysfunction using a commercially available ELISA kit (R&D Systems, Minneapolis, MN, USA). Further, the protein-bound uremic toxins, PCS and IS, were detected using a tandem mass spectrometer (Thermo Finnigan TSQ Quantum Ultra Mass Spectrometer; Thermo Fisher Scientific Inc., Waltham, MA, USA). The detailed methodology has been described in a previous study [26].

### 2.3. Statistical Analysis

**Figure 1:**
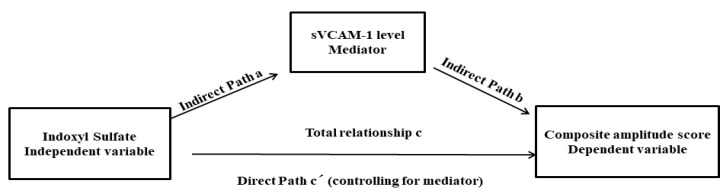
The diagram of the mediation hypothesis framework.

Data are expressed as mean ± SD or median (interquartile range). Continuous variables between two patient groups (CKD and CKD with diabetes) were compared using the independent *t*-test. Categorial data were compared by the mean of the Chi-square test or Fisher’s exact test. Those continuous variables that were not normally distributed were logarithmically transformed to improve normality and then compared using the independent *t*-test. Correlation analysis was used to evaluate the relationship between CAS and variables, including baseline cardiometabolic risk factors and biomarkers. Finally, those factors that were significantly correlated with the CAS value were enrolled into the multiple linear regression analysis models to evaluate the influence on the CAS. The protein-binding uremic toxin (IS) is a well-known uremic endotheliotoxin [27]. In this study, a single-level three-variable mediation model [28], illustrated in Figure 1, was used to investigate the causal relationships between endothelial dysfunction (sVCAM-1, mediating variable), uremic toxin (IS, independent variable), and severity of peripheral nerve dysfunction (CAS, dependent variable). The mediation analysis is employed to test whether the direct effect of an independent variable on a dependent variable can be an indirect influence through a mediator variable. The statistical significance in the Sobel test was set at *p* < 0.05 [29]. All statistical analyses were conducted using the statistical software SPSS (v26, IBM, Armonk, NY, USA).

## 3. Results

### 3.1. Baseline Characteristics of the Patients 

Of the 54 patients with CKD, 27 were non-diabetic (mean age = 67.0 years), and 27 had diabetes (mean age = 69.7 years). The baseline characteristics, underlying diseases, and cardiometabolic parameters are presented in Table 1. Regarding the underlying diseases, hypertension was the most frequent underlying disease, followed by hyperlipidemia, among both groups. The etiologies in non-diabetic CKD included chronic glomerulonephritis in 22, gouty nephropathy in three, and polycystic kidney disease in the remaining two. The biochemical parameters were similar between the two groups. The baseline peripheral blood studies and levels of biomarkers of oxidative stress, endothelial dysfunction, and protein-bound uremic toxin of patients with CKD are listed in Table 2.

### 3.2. Clinical Score, NCS, and ESC in CKD 

Clinical scores, NCS, and ESC in patients with CKD are listed in Table 3. TCNS and CAS were higher in the CKD with diabetes group than in the non-diabetic CKD group (*p* = 0.02 and 0.01, respectively). Concerning the parameters of the NCS study, the amplitude, including ulnar and peroneal CMAPs, and ulnar and sural SNAPs (*p* = 0.004, *p* = 0.004, *p* = 0.004, and *p* = 0.001, respectively) were significantly low in the CKD with diabetes group. The velocity, including ulnar, peroneal, and tibial MNCV, and median and sural SNCV, were significantly low in the CKD with diabetes group (*p* = 0.02, *p* = 0.001, *p* = 0.01, *p* = 0.04, and *p* = 0.04, respectively).

### 3.3. Effect of Protein-Bound Uremic Toxin and Cardiometabolic Risk Factors on Composite Amplitude Scores in Patients with CKD

**Figure 2:**
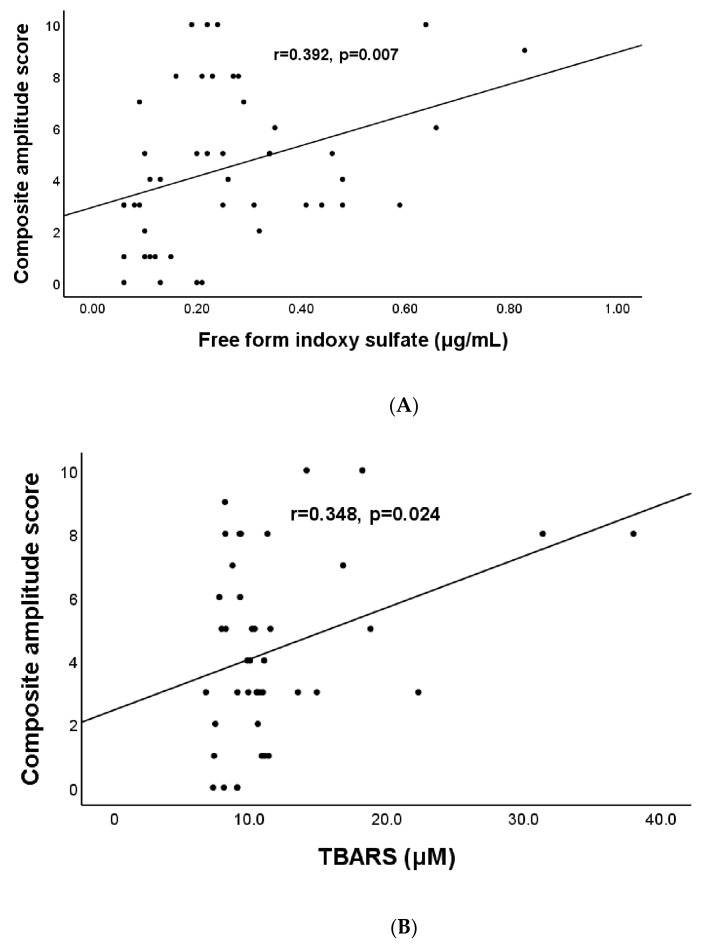
Relationship between composite amplitude score and indoxyl sulfate (**A**) and between composite amplitude score and TBARS (**B**) in patients with chronic kidney diseases.

The results of the correlation analysis used to test the influence of protein-bound uremic toxin and cardiometabolic risk factors on CAS are listed in Table 4. The statistical results (correlation coefficient, P-value) were as follows: age (year) (r = 0.27, *p* = 0.04), UACR (mg/g) (r = 0.29, *p* = 0.046), free-form IS (μg/mL) (r = 0.39, *p* = 0.007) (Figure 2A), sICAM-1 (ng/mL) (r = 0.31, *p* = 0.02), sVCAM-1 (ng/mL) (r = 0.44, *p* < 0.0001), TBARS (μmol/L) (r = 0.35, *p* = 0.002) (Figure 2B), and thiols (μmol/L) (r = −0.28, *p* = 0.045).

### 3.4. Clinical Factors Are Significantly Associated with Composite Amplitude Scores in Patients with CKD

The effects of the risk factors on CAS in patients with CKD relating to the correlation analysis are listed in Table 5. Our analysis revealed that age (year), UACR (mg/g), free-form IS (μg/mL), sICAM-1 (ng/mL), sVCAM-1 (ng/mL), TBARS (μmol/L), and thiols (μmol/L) were significantly correlated with CAS (Table 4). We employed multiple linear regression analysis to identify variables of the crucial determinant that underlie the augmented CAS in patients with CKD. The multiple linear regression analysis in the stepwise procedure found that free-form IS and TBARS were independently associated with mean CAS in the patients with CKD.

### 3.5. Mediation Analysis for Uremic Toxin (IS), the Severity of Peripheral Nerve Dysfunction (CAS), and Endothelial Dysfunction (sVCAM-1 Level)

The mediation analysis is employed to test whether the effect of uremic toxin (IS, independent variable) on the severity of peripheral nerve dysfunction (CAS, dependent variable) was influenced by sVCAM-1 level (mediator) indirectly. The path model jointly tested three effects: (a) the effect of IS value (independent variable) on the sVCAM-1 level(mediator) (indirect effect, path a); (b) the effect of the sVCAM-1 level (mediator) on the CAS (dependent variable) (indirect effect, path b); and (c) the mediation effect a × b, which is defined as the reduction of the relationship between the IS and CAS (independent and dependent variables) (total relationship, path c) by including the sVCAM-1 (mediator) into the model (direct path, path c′). The mediation relationship was significant (*p* = 0.017 in the Sobel test) (Table 6).

## 4. Discussion

### 4.1. Major Findings of Our Study

Although indoxyl sulfate is an endotheliotoxin and is involved in the pathophysiology of cardiovascular events in CKD [27,30,31], there is a paucity of information located on the novelty of peripheral nerve dysfunction. This is the first study to show that IS contributes to the severity of peripheral nerve function in patients with CKD and that chronic glycemic impairment can further worsen the condition. Our study also highlighted the hypothesis that endothelial dysfunction mediates the relationship between uremic toxin (IS) and the severity of peripheral nerve function in patients with CKD. Besides protein-binding uremic toxin (IS) as a uremic endotheliotoxin [27], oxidative stress might serve to drive the process.

### 4.2. The Pathophysiology of Protein-Binding Uremic Toxins 

Concerning biochemical and physical properties, uremic toxins can be divided into three groups as follows: water-soluble, non-protein-binding, low molecular weight compounds (e.g., urea and creatinine); larger or medium molecular weight compounds (e.g., 2-microglobulin); and protein-binding low molecular weight compounds (e.g., IS and PCS) [8]. Among them, protein-binding uremic toxins are difficult to remove by dialysis procedures because of their high protein-binding capabilities [8]. When tryptophan is present in food, intestinal bacteria (mainly *Escherichia coli*) can metabolize tryptophan to indole. Indole is absorbed by the intestine, circulates in the blood to the liver, undergoes hydroxylation and sulfation by the liver, becomes IS, and finally re-enters the blood circulation. PCS, another protein-binding uremic toxin, is a product of the metabolism of tyrosine and phenylalanine by the intestinal bacterium that is excreted by the kidneys [32].

### 4.3. The Potential Pathogenesis of Protein-Binding Uremic Toxins in Peripheral Nerve Function 

The clinical impacts of IS levels on cardiac and renal development have been well-documented [32,33,34]. In contrast, the clinical impact of IS in peripheral nerve injury has rarely been studied. Although the pathogenesis of polyneuropathy could be complex and multifactorial, oxidative stress and endothelial dysfunction may be involved in this pathogenesis. Some studies support the view that exposure of endothelial cells to IS increases the formation of free radicals. IS also induces ROS production in endothelial cells [35]. The accumulation of free radicals can trigger a series of responses, including proliferation arrest, inducing apoptosis of cells, and impairing neovascularization. Free radical production also triggers oxidative stress, enhances the activity of NF-kB and tissue inhibitor matrix metalloproteinase 1, enhances the expression of cytokines and inflammatory responses, and causes cell damage. ICAM-1 and VCAM-1 are regulated by NF-kB [36]. The previous study demonstrated the IS increased ICAM-1 and VCAM-1 expression [37] and induced increases in the levels of cell adhesion molecules, such as ICAM-1, VCAM-1, and E-selectin to activation and adhesion of leukocytes to the endothelium [38]. Oxidative stress and expression of the VCAM-1 on vascular endothelial cells are early features in the pathogenesis of atherosclerosis and other inflammatory diseases such as diabetes mellitus and chronic kidney disease [39]. The previous study suggests the potential role of plasma cell adhesion molecules in the pathogenesis of diabetic neuropathy [40]. Another study concluded the plasma VCAM-1 in diabetic patients with microalbuminuria was 1.5-fold greater compared to diabetic patients without microalbuminuria [41]. Furthermore, accumulating evidence indicates that chronic persistent hyperglycemia has a negative effect on various pathogenesis in diabetes [42,43]. Both IS and hyperglycemia could enhance oxidative stress and the resultant downstream regulation of signaling pathways (e.g., NF-kB), contribute to both endothelial microangiography (ischemia) and cell dysfunction, and, ultimately, result in increased severity of peripheral nerve dysfunction. This is the reason that patients with diabetes are prone to more severe peripheral nerve function than those with non-diabetic CKD [42,43].

### 4.4. Electrophysiological Parameters and Clinical Scores in Patients with CKD

One recent large study enrolled 200 patients in the pre-dialysis stage, including 100 patients with CKD with diabetes and 100 other non-diabetic patients with CKD, and found that the prevalence of polyneuropathy was 45%. It was also noted that the parameters of the NCS were more severe in the CKD with diabetes subgroup in comparison with the non-diabetic CKD subgroup [44]. The severity of the NCS in their study was found to be similar to that of our study. Another study enrolled 40 patients (20 patients with pre-dialytic CKD and 20 with CKD with diabetes) and found that the clinical neuropathy score and parameters of NCS, including tibial and median CMAPs and sural SNAPs, were worse in the CKD with diabetes subgroup [45]. Our study further showed the severity of peripheral nerve dysfunction, demonstrated by both TCNS and CAS, was worse in the CKD with diabetes subgroup. The parameters of NCS, including amplitude and velocity, in most sensory and motor nerves were significantly lower in CKD with the diabetes subgroup in comparison with the nondiabetic CKD subgroup. It could be debated that only CAS may not reflect the severity of peripheral nerve function of nerve conduction. Our recent study used CAS (point-based method) as the measurement of the severity of nerve conduction in type 2 diabetes mellitus (T2DM) [22] because peripheral neuropathy in both CKD and diabetes mainly follows axonal pathophysiology [12,46]. Therefore, CAS could be valid in the measurement of the severity of peripheral nerve dysfunction.

### 4.5. Study Limitations

This study has four limitations. First, this was a prospective cross-section observational study. Although our results showed that protein-binding uremic toxin, oxidative stress, and endothelial dysfunction might serve to drive the process of the severity of peripheral nerve dysfunction, a causal link connecting protein-binding uremic toxin, oxidative stress, and endothelial dysfunction with the severity of peripheral nerve function in CKD remains to be established. Second, patients with diabetes are prone to more severe peripheral nerve impairment than those without diabetes; however, the exact differences in pathogenesis in peripheral neuropathy between these two subgroups also should be clarified. A cell culture study showed that dipeptidyl peptidase-4 (DPP-4) inhibitors possess anti-apoptotic activity to ameliorate the IS-induced renal damage, which may be via regulating the ROS/p38MAPK/ERK and PI3K-AKT pathways as well as the downstream NF-kB signaling pathway [47]. Our study showed that IS contributes to the severity of peripheral nerve dysfunction, and our recent study also showed a close relationship between the severity of kidney damage and peripheral nerve dysfunction in patients with T2DM [6]. The sample size of our study was small, and we did not investigate the role of oral hypoglycemic agents. If the DPP-4 inhibitors possess anti-IS-induced cell damage activity, they may ameliorate both renal and peripheral nerve function in patients; this needs to be further explored in a longitudinal study. Finally, oral adsorbents (e.g., AST-120) [10] adsorbed the precursors of IS and PCS generated by amino acid metabolism in the intestine, and reduced serum protein-binding uremic toxin (e.g., IS and PCS) levels could ameliorate both renal and peripheral nerve function and need to be assessed in a longitudinal study.

## 5. Conclusions

The proteinuria-binding uremic toxin IS, but not PCS, contributes to the severity of peripheral nerve dysfunction in patients with CKD, and chronic glycemic impairment can worsen the condition. Besides protein-binding uremic toxin, oxidative stress might serve to drive the process.

## Figures and Tables

**Table 1:** Baseline characteristics of patients with diabetic and non-diabetic chronic kidney diseases.

	Diabetic CKD (n = 27)	Non-Diabetic CKD (n = 27)	*p*-Value
Baseline characteristics			
Age (year)	69.7 ± 12.4	67.0 ± 10.2	0.22
Sex (male/female)	16/11	18/9	0.57
Diabetes duration (year)	16.2 ± 11.1	-	
Height (cm)	163.3 ± 8.0	161.5 ± 8.3	0.42
Body mass index	26.5 ± 5.4	26.5 ± 5.4	0.11
Waist circumstance (cm)	96.8 ± 14.8	90.0 ± 10.3	0.10
Systolic blood pressure (mmHg)	140.5 ± 23.2	151.9 ± 25.6	0.09
Diastolic blood pressure (mmHg)	75.3 ± 14.9	81.9 ± 16.5	0.13
ACE inhibitor or angiotensin II receptor blocker	20	22	0.51
Beta-blocker	13	13	1.0
Calcium channel blocker	18	14	0.27
Diuretics	8	1	0.02
Alpha-blocker	4	3	1.0
Antiplatelet medications	13	9	0.27
Lipid-lowering medications	22	15	0.04

Data are presented as means ± standard deviations or n (%). Abbreviations: n, number of cases; CKD = chronic kidney disease; Only seven patients received monotherapy, and one did not receive the antihypertensive drug.

**Table 2:** Baseline biochemical data of patients with diabetic and non-diabetic chronic kidney diseases.

	Diabetic CKD (n = 27)	Non-Diabetic CKD (n = 27)	*p*-Value
Peripheral blood studies			
WBC counts (×10^3^/mL)	7.5 ± 2.5	6.0 ± 1.6	0.02
RBC counts (×10^6^/mL)	4.0 ± 0.6	4.0 ± 1.0	0.93
Hemoglobin, g/dL	12.0 ± 1.7	12.0 ± 2.1	0.96
Hematocrit	36.0 ± 4.8	36.0 ± 6.8	0.98
Platelet counts (×10^3^/mL)	236.2 ± 55.1	195.7 ± 55.5	0.02
UACR (mg/g)	258.9 (78.3, 1052.5)	276.6 (18.0, 758.6)	0.43
eGFR (mL/min/1.73 m^2^)	31.2 ± 9.8	30.6 ± 11.4	0.08
Creatinine (mmol/L)	2.0 ± 0.8	2.3 ± 0.7	0.1
Albumin (mg/dL)	4.5 ± 0.3	4.6 ± 0.3	0.49
Total cholesterol l(mmol/L)	160.9 ± 38.5	183.3 ± 57.8	0.11
Triglyceride (mmol/L)	117.5 ± 65.3	118.2 ± 53.7	0.97
HDL-C (mmol/L)	45.5 ± 11.0	47.5 ± 13.4	0.56
LDL-C (mmol/L)	88.3 ± 30.6	111.2 ± 44.8	0.03
Glycohemoglobin (%)	6.9 ± 1.1	5.5 ± 0.3	<0.0001 *
Uric acid (mg/dL)	6.6 ± 1.9	6.9 ± 1.5	0.57
Calcium (mmol/L)	9.3 ± 0.4	9.4 ± 0.4	0.48
hs-CRP, mg/L	2.2 ± 1.4	1.7 ± 1.0	0.47
Potassium (mmol/L)	4.5 ± 0.8	4.5 ± 0.7	0.91
Sodium (mmol/L)	139.7 ± 4.3	139.4 ± 3.5	0.78
Phosphate (mmol/L)	3.7 ± 0.8	3.6 ± 0.6	0.65
CO_2_	29.2 ± 22.7	31.7 ± 24.6	0.74
iPTH (pg/mL)	125.5 ± 87.8	156.3 ± 94.0	0.6
Biomarkers for endothelial dysfunction			
sICAM-1 (ng/mL)	242.2 ± 69.1	245.1 ± 29.6	0.93
sVCAM-1 (ng/mL)	1083.0 ± 259.5	971.1 ± 94.0	0.36
Biomarkers for oxidative stress			
TBARS, μmol/L	13.4 ± 7.9	9.9 ± 2.6	0.09
Thiols, μmol/L	1.0 ± 0.5	1.1 ± 0.4	0.57
Protein-bound uremic toxin			
Free-form Indoxyl sulfate (μg/mL)	0.24 ± 0.17	0.21 ± 0.15	0.54
Free-form p-Cresol sulfate (μg/mL)	0.50 ± 0.49	0.45 ± 0.41	0.73
Total-form Indoxyl sulfate (μg/mL)	4.0 ± 3.3	3.9 ± 2.3	0.88
Total-form p-Cresol sulfate (μg/mL)	11.6 ± 10.4	10.4 ± 9.8	0.65

Data are presented as means ± standard deviations or median (interquartile range). * Indicates that *p*-value < 0.05. Abbreviations: n, number of cases; DKD = diabetic kidney disease; CKD = chronic kidney disease; eGFR = estimated glomerular filtration rate; UACR = urine albumin-creatinine ratio; HDL-C: High-density lipoprotein cholesterol; LDL-C: Low-density lipoprotein cholesterol; iPTH = parathyroid hormone; TBARS, thiobarbituric acid-reactive substance; sICAM-1, serum intercellular adhesion molecule 1; sVCAM-1, serum vascular adhesion molecule 1.

**Table 3:** Clinical score and the results of the nerve conduction study and electrochemical skin conductance between patients with diabetic and non-diabetic chronic kidney diseases.

	Diabetic CKD (n = 27)	Non-Diabetic CKD (n = 27)	*p*-Value
Toronto Clinical Neuropathy Score	5.7 ± 4.1	3.4 ± 3.0	0.02 *
Composite amplitude score	5.3 ± 3.2	3.2 ± 2.8	0.008 *
Median nerve, motor			
DML	4.4 ± 0.6	4.1 ± 0.7	0.12
CMAP	8.5 ± 2.1	9.6 ± 2.9	0.12
MNCV	51.1 ± 4.3	52.9 ± 4.2	0.12
Ulnar nerve, motor			
DML	3.1 ± 0.4	3.0 ± 0.4	0.17
CMAP	7.9 ± 2.3	9.8 ± 2.6	0.004 *
MNCV	51.3 ± 5.5	54.6 ± 4.9	0.02 *
Peroneal nerve,			
DML	4.1 ± 0.6	3.8 ± 0.6	0.03 *
CMAP	2.4 ± 1.8	4.2 ± 2.7	0.004 *
MNCV	41.4 ± 4.3	46.0 ± 5.1	0.001 *
Tibial nerve			
DML	4.2 ± 0.6	4.0 ± 0.5	0.06
CMAP	7.4 ± 5.3	8.7 ± 4.3	0.29
MNCV	41.3 ± 5.3	44.5 ± 3.3	0.01 *
Median nerve, sensory			
Latency	3.3 ± 0.4	3.1 ± 0.5	0.04 *
SNAP	23.2 ± 13.2	30.4 ± 15.6	0.06
SNCV	42.6 ± 5.8	46.2 ± 7.8	0.04 *
Ulnar nerve, sensory			
Latency	2.6 ± 0.4	2.4 ± 0.3	0.04 *
SNAP	17.8 ± 12.2	27.7 ± 13.5	0.004 *
SNCV	46.8 ± 6.4	49.8 ± 5.3	0.07
Sural nerve			
Latency	3.1 ± 0.4	2.9 ± 0.3	0.07
SNAP	4.0 ± 2.7	9.9 ± 7.0	0.001 *
SNCV	44.8 ± 4.8	48.3 ± 5.3	0.04 *
Sudoscan			
Hand ESC, µS	40.6 ± 19.7	45.7 ± 18.5	0.30
Feet ESC, µS	44.4 ± 18.2	48.4 ± 23.6	0.54

Data are presented as means ± standard deviations or n (%). * Indicates that *p*-value < 0.05. Abbreviations: n, number of cases; DML = distal motor latency; CMAP = compound muscles action potential; MNCV = motor nerve conduction velocity; SNAP = sensory nerve action potential; SNCV = sensory nerve conduction velocity; ESC, electro-chemical skin conductance.

**Table 4:** Correlation analysis of composite amplitude score on cardiometabolic parameters in patients with diabetic and non-diabetic chronic kidney diseases.

Variables	Composite Amplitude Scores	
	r	*p*-Value
Age (year)	0.27	0.04 *
Height (cm)	0.18	0.23
Body mass index	0.12	0.43
Waist circumstance (cm)	0.14	0.39
eGFR (mL/min/1.73 m^2^)	−0.008	0.96
UACR (mg/g)	0.29	0.046 *
Free-form Indoxyl sulfate (μg/mL)	0.39	0.009 *
Free-form p-Cresol sulfate (μg/mL)	0.26	0.10
Total-form Indoxyl sulfate (μg/mL)	0.28	0.07
Total-form p-Cresol sulfate (μg/mL)	0.31	0.05
sICAM-1 (ng/mL)	0.31	0.02 *
sVCAM-1 (ng/mL)	0.44	<0.0001 *
TBARS, μmol/L	0.35	0.002 *
Thiols, μmol/L	−0.28	0.045 *
Total cholesterol(mmol/L)	−0.19	0.22
Triglyceride(mmol/L)	−0.20	0.18
HDL-C (mmol/L)	−0.16	0.28
LDL-C (mmol/L)	−0.08	0.6
Uric acid (mmol/L)	−0.04	0.8
hs-CRP (mmol/L)	0.18	0.3
HbA1c (%)	0.04	0.82

r: correlation coefficient. * Indicates that *p*-value < 0.05. Abbreviations: HDL-C, high-density lipoprotein cholesterol; LDL-C, low-density lipoprotein cholesterol; UA, uric acid; hsCRP, high-sensitive C-reactive protein; HbA1c, glycohemoglobin; eGFR, estimated glomerular filtration rate; UACR = urine albumin-creatinine ratio TBARS, thiobarbituric acid-reactive substance; sICAM-1, serum intercellular adhesion molecule 1; sVCAM-1, serum vascular adhesion molecule 1.

**Table 5:** Effects of the variables on composite amplitude scores in patients with diabetic and non-diabetic chronic kidney diseases according to correlation analysis.

	Model		
	Regression Coefficient	Standard Error	*p*-Value
Constant	0.56	0.94	0.56
TBARS, μmol/L	0.17	0.06	0.007
Indoxyl sulfate (μg/mL)	5.04	2.65	0.035

The regression coefficient for each individual variable. Abbreviation: TBARS, thiobarbituric acid-reactive substance. Model: R2 = 0.493. Predictors in the model: constant, TBARS, Indoxyl sulfate.

**Table 6:** A simple mediation model of uremic toxin (Indoxyl Sulfate [X]) on the severity of Peripheral Nerve Dysfunction (Composite amplitude scores [Y]) through endothelial dysfunction (sVCAM-1 [M]) effort.

	Path Coefficient	Standard Error	*p*-Value
Total effects (total relationship, path c) ^Ω^			
The relationship between the IS (independent variable) and CAS (dependent variable)	7.87	2.50	0.003
Direct effects, path c′			
The relationship between the IS (independent variable) and CAS (dependent variables) by including the sVCAM-1 (mediator) into the model	5.06	2.56	0.45
Indirect effect, path a			
The effect of the IS (independent variable) on the sVCAM-1 (mediator)	704.08	235.26	0.004
Indirect effect, path b			
The effect of the sVCAM-1 (mediator) on the CAS (dependent variable by controlling the effect for the IS (independent variable)	0.004	0.001	0.01

Abbreviations: X = Indoxyl Sulfate (independent variable); Y = severity of peripheral nerve dysfunction: (dependent variable); M = sVCAM-1 level(mediator). Ω = The mediation effects a × b which is defined as the reduction of the relationship between the independent and dependent variables (uremic toxin-severity of peripheral nerve dysfunction) (total relationship, path c) by including the mediator into the model (direct path, path c′); (Sobel test, *p* = 0.017).

## Data Availability

Data is contained within the article.

## Abbreviations

eGFR: estimated glomerular filtration rate; PN: peripheral neuropathy; DSPN: diabetic sensorimotor polyneuropathy; SBP: systolic blood pressure; DBP: diastolic blood pressure; DR: diabetic retinopathy; UACR: urinary albumin-to-creatinine ratio; NCS: nerve conduction studies; NCV: nerve conduction velocity; BMI: body mass index; CMAP: compound muscle action potential; SNAP: sensory nerve action potential; MNCV: motor nerve conduction velocity; SNCV: sensory nerve conduction velocity.
